# Patterned Cell Adhesion Associated with Tissue Deformations during Dorsal Closure in *Drosophila*

**DOI:** 10.1371/journal.pone.0027159

**Published:** 2011-11-04

**Authors:** Ana Margarida Mateus, Alfonso Martinez Arias

**Affiliations:** 1 Department of Genetics, University of Cambridge, Cambridge, United Kingdom; 2 Gulbenkian PhD Programme in Biomedicine, Oeiras, Portugal; National Institutes of Health (NIH), United States of America

## Abstract

Cell shape changes within epithelia require the regulation of adhesive molecules that maintain tissue integrity. How remodelling of cell contacts is achieved while tissue integrity is maintained remains a fundamental question in morphogenesis. Dorsal Closure is a good system to study the dynamics of DE-Cadherin during morphogenesis. It relies on concerted cell shape changes of two epithelial sheets: amnioserosa cell contraction and epidermal cell elongation. To investigate the modulation of DE-Cadherin we performed antibody uptake experiments in live embryos during Dorsal Closure. We found that some antibodies access certain epitopes of the extracellular domain of native DE-Cadherin only in the amnioserosa and epidermal cells attached to the amnioserosa, which has never been observed in fixed DE-Cadherin in *Drosophila* embryos. These differences correlate with the different cell behaviour of these regions and therefore we suggest that DE-Cadherin exists in different forms that confer different adhesive strengths. We propose this to be a widespread mechanism for the differential modulation of adhesion during morphogenesis.

## Introduction

The Cadherin protein family is a group of calcium dependent homophilic cell adhesion molecules that mediate adhesion between cells [1]. The signature of this protein family is an extracellular domain composed of “cadherin domains” that promote intercellular interactions, and an intracellular domain that serves as a link between the intercellular adhesion and the actin cytoskeleton through interactions with the catenins [1]. In epithelia, Cadherins localise at the Adherens Junctions (AJs) near the apical side of the cell and generate a continuum between the actin cytoskeleton of different cells allowing coordinated tissue deformation [2], [3], [4]. Although the dynamics of cytoskeletal activity during morphogenesis is being extensively studied [5], less is known about how adhesion is modulated during these processes. Biophysical models of morphogenetic processes predict that changes in adhesion are important in the modulation of the mechanical properties of epithelia [6]. This could be achieved by modulating the total amount of Cadherin, through the regulation of its expression [7], [8], [9], [10], or its steady-state levels at the membrane, through endocytosis and recycling [11], [12]. A third mechanism could target the adhesive properties of Cadherin, regulating its conformation, clustering state and other higher-order organizations [1].

Assessment of Cadherin adhesive properties *in vivo* during morphogenesis is difficult since genetic removal of Cadherin has a dramatic effect on tissue integrity. Dorsal Closure (DC) in *Drosophila* represents a good model to address DE-Cadherin modulation *in vivo*. DC is a process whereby two epithelia, the epidermis and the amnioserosa (AS), interact to cover a discontinuity on the dorsal epidermis of the *Drosophila* embryo [13], [14]. It is associated with cell shape changes and local cell interactions as generators of dynamical force fields that drive a patterned contraction of the AS and a correlated epidermis elongation [15], [16], [17]. *Drosophila* E-Cadherin, DE-Cadherin, encoded by the *shotgun* (*shg*) gene, provides an essential cell adhesion force, balancing the stresses generated during DC [18]. Removal of DE-Cadherin, both maternally and zygotically, results in the loss of epithelial integrity early in embryogenesis [10], [19]. However zygotic null mutants for *shg* receive maternal DE-Cadherin that allows the embryos to initiate DC with reduced levels of DE-Cadherin levels [18]. Interestingly, embryos mutant for null alleles of *shg* are rescued by ubi-DE-CadherinGFP expression and develop into normal adult flies [20] suggesting that any modulation of Cadherin activity during development might occur at the post-transcriptional level.

Here we investigate post-transcriptional modulations in DE-Cadherin during a morphogenetic process. Our study reveals surprising spatial differences in the configuration of the extracellular domain of DE-Cadherin which correlate with patterned cell shape changes during DC. We propose that these differences represent Cadherins with different adhesive properties.

## Materials and Methods

### Drosophila strains

Wild-type embryos were from the Oregon R strain, *w ; shg^R64^/CyO* strains (Tepass et al., 1996), ubi-DE–CadherinGFP [20], *Shg^R64^* homozygous mutant embryos were selected from a cross between *w; shg^R64^, UASactinGFP/CyO* and *w; shg^R64^, enGal4/CyO* (N. Gorfinkiel).

### *In vivo* hand-devitellinization

Our hand-devitillinization protocol follows published reports [21]. Embryos at DC stage were selected and aligned with the ventral region upward and anterior part towards the observer on top of a narrow stripe of double-sided tape. Sörensen phosphate buffer (SPB) was added to cover the aligned embryos. The vitelline membrane was pierced at the head with a glass needle that was moved to the posterior of the embryo; the movement is done without indenting deep in the embryo. The embryo was teased out of the vitelline membrane, away from the tape.

### Antibody uptake assays

Hand-devitellinized embryos were transferred with a coated glass pipette into a coated glass dish with SPB at 4°C, then to another glass dish with 500 µl of cold SPB containing primary antibodies and incubated for 1 hour at 4°C, rinsed 3 times and finally washed 6 times for 2 minutes with SPB at 4°C. The embryos were either immediately fixed (time 0) or chased for 10, 30 minutes or 1 hour in Schneider's insect medium supplemented with 10% Fetal Calf Serum (FCS) and 1% L-Glutamine at 25°C. Fixation was performed in paraformaldehyde (PFA) 4% for 40 minutes at 25°C, wash-blocked (3 rinses plus four 10 minutes incubations) in BBT-BSA (BBS + CaCl_2_ 1 mM + 0,1% Triton + 0,5% BSA). For further antibody labelling, embryos were incubated with other primary antibodies diluted in BBT-BSA for 2 hours at Room Temperature (RT), and thoroughly washed with BBT-BSA. Finally, embryos were incubated with 500 µl of BBT-BSA containing secondary antibodies at RT for 2 hours in the dark, rinsed 3 times and washed 4 times in BBT-BSA and then individually mounted in Vectashield.

The pulse-chases were done simultaneously, with ±6 embryos for each time point. The experiment was repeated 3 times. Thereafter all the experiments with different antibodies or mutant embryos were done using the same protocol without chase, always using DCAD2 as a control.

### Antibodies

The following primary antibodies were used: rat anti-DE-Cadherin DCAD1 (T. Uemura) 1∶100, rat anti-DE-Cadherin DCAD2 (DSHB) 1∶200, rabbit anti-DE-Cadherin d-300 (Santa Cruz) 1∶100, goat anti-DE-Cadherin-intra dP-20 (Santa Cruz) 1∶200, rat anti-DE-Cadherin (V. Hartenstein) 1∶50, mouse anti-Notch-extra C458.24 (DSHB) 1∶50, rabbit anti-Scribble (C. Doe) 1∶1000, 1∶10. Secondary antibodies were from Molecular Probes.

### Immunostainings

Embryos were fixed and stained as previously described (Kaltschmidt et al., 2002). Fluorescently labelled embryos were mounted in Vectashield (Vector) and examined under a Nikon D-Eclipse C1 confocal scanning unit, mounted on a Nikon Eclipse 90i microscope, using the EZ-C1 3.60 software and a 60x/1.40 NA Apo VC oil-immersion objective. Five-seven z-sections, 0.5 µm apart, were projected using ImageJ (http://rsb.info.nih.gov/ij/) and processed using Photoshop.

### Quantification of fluorescence intensities

For quantification of fluorescence intensity, the polygon selection tool was used to draw around an object and the mean gray value was obtained using ImageJ. Notch and DCAD2 fluorescence intensity from different time points were compared using One-Way ANOVA and Tukey HSD Test for Post-ANOVA Pair-Wise Comparisons.

### Time-lapse movies

**Figure 1:**
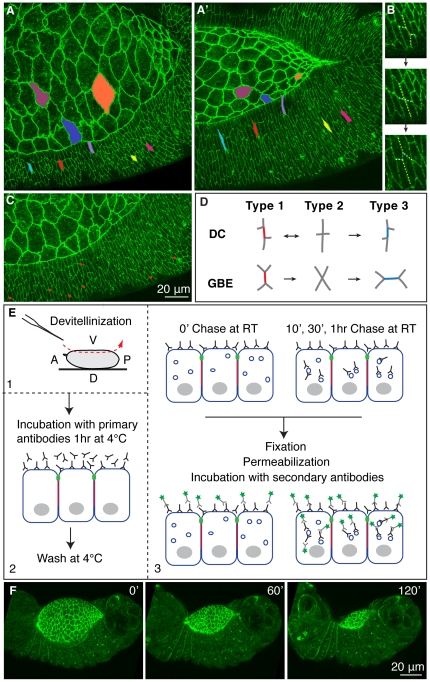
Cell shape changes and exchange of cell neighbours in DC. (A–C) Stills from a time-lapse of an Ubi-DE-CadherinGFP embryo during DC. Colours identify cells that are followed during the process, highlighting cell shape changes (A,A′). Exchanges between neighbours (B) in the lateral epidermis (C). The number of cell neighbour exchanges is 6.4 ± 1.2 per segment (first 6 epidermal rows in 5 segments in 3 embryos over 90 minutes). (D) Pattern of neighbour exchange during DC and GBE. (E) Overview of the pulse-chase assay (antibodies appear outside the vesicles for simplicity). (F) Stills from a time-lapse of a hand-devitellinized Ubi-DE-CadherinGFP embryo (Movie S1).

Stage 13 *Drosophila* embryos carrying an ubi-DE-CadherinGFP construct (Oda and Tsukita, 2001), were dechorionated, mounted on coverslips with the dorsal side glued to the glass and covered with Voltalef oil 10S (Attachem). Imaging of the embryos was done using an inverted LSM 510 Meta laser-scanning microscope with a Plan-Apochromat 63x/1.4 oil-immersion objective. Embryos were maintained at 24°C during imaging and around 50 z-sections 1 µm apart were collected every 2 minutes. For Figure 1F, Ubi-DE-CadherinGFP *Drosophila* embryos at stage 13 were dechorionated, devitellinized and transferred into small dips in agar glass dishes with insect medium and imaged every 2 minutes, collecting around 50 z-sections 1 µm apart under a Nikon D-Eclipse C1 confocal scanning unit, mounted on a Nikon Eclipse 90i microscope, using the EZ-C1 3.60 software and a Nikon Fluor 40x10.80 water-immersion objective. Movies were assembled and processed ImageJ (http://rsb.info.nih.gov/ij/).

## Results and Discussion

During DC, contraction of the AS cells provides a tensile force that drives and maintains elongation of the epidermal epithelium [13], [15], [16], [22] (Figure 1A,A′). During epidermal elongation the average ratio between the Dorsal/Ventral (D/V) and Anterior/Posterior (A/P) cell axis changes from 1.5 to 5.2 (Young et al., 1993). There are two well defined domains in the epidermis: the Dorsal Most Epidermal (DME) cells, which form an interface between the AS and the epidermal sheet and bear the brunt to the forces of the process, and the lateral epidermis, where the elongation is associated with local cell rearrangements. Analysis of these rearrangements in the first 6 epidermal rows of 5 segments in 3 embryos over the period of 90 minutes reveals stereotyped exchange of neighbours (Figure 1B,C and Movie S1) and a number of 6.4 ± 1.2 cell neighbour exchanges per segment. During Germ Band Elongation (GBE) [23], [24] cells also undergo a sequence of cell contact changes termed T1>T2>T3 transition (Figure 1D,[23]). However, while in GBE the process is continuous and irreversible, T1>T2 type transitions are frequently maintained or reversed during DC, not leading to cell intercalation (Figure 1D). This difference might result from the fact that in GBE cell contact changes underlie tissue elongation that is driven by local forces [25] whereas in DC there is an external pulling force generated by AS contraction that drives epidermal cell elongation [15], [16], [17] and these transitions accommodate stresses associated with cell elongation in relation to neighbours. A similar conversion of junctions has been observed in the ventral epidermis of earlier *Drosophila* embryos during epidermal cell alignment along the D/V boundary [26] suggesting that they represent a theme in morphogenesis [24].

Cell shape changes and exchange of neighbours require modulation of the cell surface molecules, in particular of DE-Cadherin [27], [28]. As there are no reports of differential expression of DE-Cadherin in the epidermis during DC, we looked for dynamic changes in the cell surface pool of DE-Cadherin by labelling and chasing this pool of DE-Cadherin. We adapted an existing culture technique that retrieves the embryo from the vitelline membrane [21], allows progression of DC (Figure 1F) and makes the cells competent to take up dyes and antibodies (Figure 1E_2_-E_3_). To test the assay, we pulse labelled embryos with antibodies against the extracellular domain of DE-Cadherin, and observed a translocation of the antibody into intracellular vesicles. In contrast, antibodies against the intracellular domain of the transmembrane receptor Notch or the intracellular protein Scribble did not show any signal (Figure S1A′,A″,B′,B″) which was only detected when cells were permeabilized before incubation (Figure S1C′,C″). These experiments validate this protocol for dynamic analysis of cell surface proteins.

### Dynamic assays reveal that monoclonal antibody binding to native DE-Cadherin is patterned

To study the dynamics of DE-Cadherin during DC, stage 13–14 Ubi-DE-CadherinGFP embryos were incubated at 4°C with the monoclonal antibody DCAD2, directed against the extracellular domain of DE-Cadherin, and with an antibody against the extracellular domain of Notch as a control, and chased for different time periods (0, 10, 30 minutes and 1 hour) at RT.

**Figure 2:**
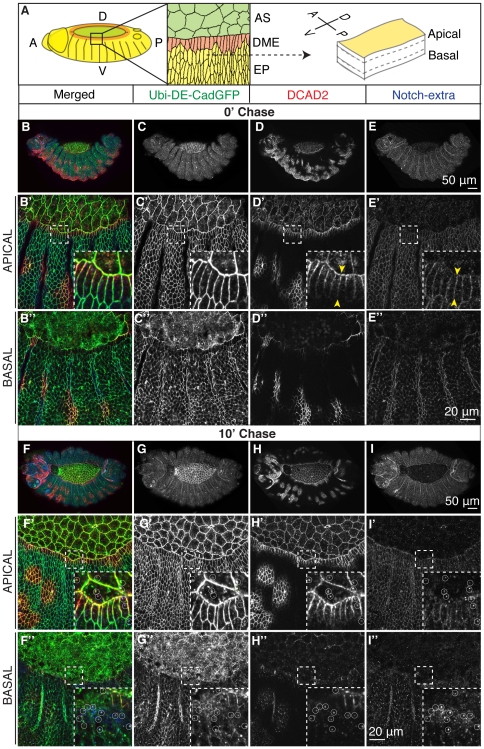
***In vivo* antibody binding to native DE-Cadherin reveals patterned access to DE-Cadherin in the cell surface.** (A) Cartoon depicting the two tissues analyzed in the assays, the AS (green) and the epidermis (EP), which comprises the DME (orange), and the lateral epidermis (yellow). (B–I) Pulse-chase assay in Ubi-DE-Cadherin-GFP embryos using DCAD2 and Notch-extra antibodies. Yellow arrows highlight the binding of DCAD2 and Notch-extra antibody along the D/V contact of DME cells.

Embryos that have been fixed immediately after antibody loading, express DE-CadherinGFP in all cells (Figure 2C,C′,C″), however the binding of the DCAD2 antibody to the epidermal cells reveals a spatial pattern: the AS and the DME exhibit clear antibody binding which is absent in the lateral epidermal cells, except in small patches (Figure 2D,D′,D″). We have not investigated the nature of these patches but they could result e.g. from local rearrangements occurring underneath the epidermis that impinge into the epidermis tension and adhesion systems. The binding of DCAD2 to the AS and DME cells mainly, could be due to an inability of the antibodies to access epitopes on the surface of lateral epidermal cells. However, under the same experimental conditions, antibodies against the extracellular domain of Notch (Figure 2E, E′,E″), bind homogeneously to all the epidermal cells. Interestingly, in the DME cells, the borders that face lateral cells have less DCAD2 labelling than the others, and we observe a gradient of label along the D/V border (Figure 2D′ inset yellow arrows). In contrast, Notch antibodies bound homogeneously along the borders of all epidermal cells (Fig 2E′ inset yellow arrows). After 10 minutes of chase the pattern of DCAD2 binding was preserved and vesicles positive for DCAD2 and DE-CadherinGFP could be detected inside DCAD2 labelled cells (Figure 2G,G′,H,H′), which indicates that the pool of DE-Cadherin recognized by DCAD2 is dynamic. Vesicles containing Notch were observed in all epidermal cells at the same chase time, showing that both proteins are endocytosed in less then 10 minutes (Figure 2I,I″). Nevertheless, after 30 minutes of chase at room temperature, differences in the localization at the membrane of both proteins were accentuated. Even though both proteins seem to be equally abundant inside the cell, the majority of Notch protein detected by the antibody is cleared from the membrane over time, which contrasts with DE-Cadherin detected by the antibody, which stays at the membrane even with longer chases, indicating that both proteins have different turnovers at the cell surface (Figure S2C′,C″,D′,D″,I). These differences probably reflect their different functions. DE-Cadherin mediates cell-cell adhesion and Notch is a signalling molecule which undergoes endocytosis as part of its signalling activity [30], [31]. In agreement with this, after 1 hour chase, labelled Notch was undetectable (Figure S2H′,H″) but DCAD2 antibody was still detected at the membrane or in large vesicles, especially at the cell basal region (Figure S2G′,G″).

It has been reported that crosslinking of cell surface proteins by antibodies might trigger endocytosis [29]. While this remains a possibility, pulse-chase assays performed with Dextran revealed that DE-Cadherin is endocytosed in the epidermis and AS with Dextran (not shown), suggesting that an important fraction of what we observe is related to DE-Cadherin endocytosis.

It is noteworthy that the differential binding of DCAD2 to epidermal cells was also observed in wild-type embryos (Figure S3E-G′) and thus it is not a consequence of the expression of DE-CadherinGFP with the wild-type protein. The pattern has been seen in wild-type embryos in 76.4% ± 16.3 of the embryos at 0′ chase (n = 67 embryos from 5 independent experiments) and in ubi-DE-CadherinGFP embryos 73.2% ± 19.9 (n = 56 embryos from 8 independent experiments). The dynamics of this pattern could not be investigated in later embryos because cuticle secretion, which begins at stage 15, interferes with antibody binding.

### Antibodies against different epitopes of DE-Cadherin bind differently along the epidermis

**Figure 3:**
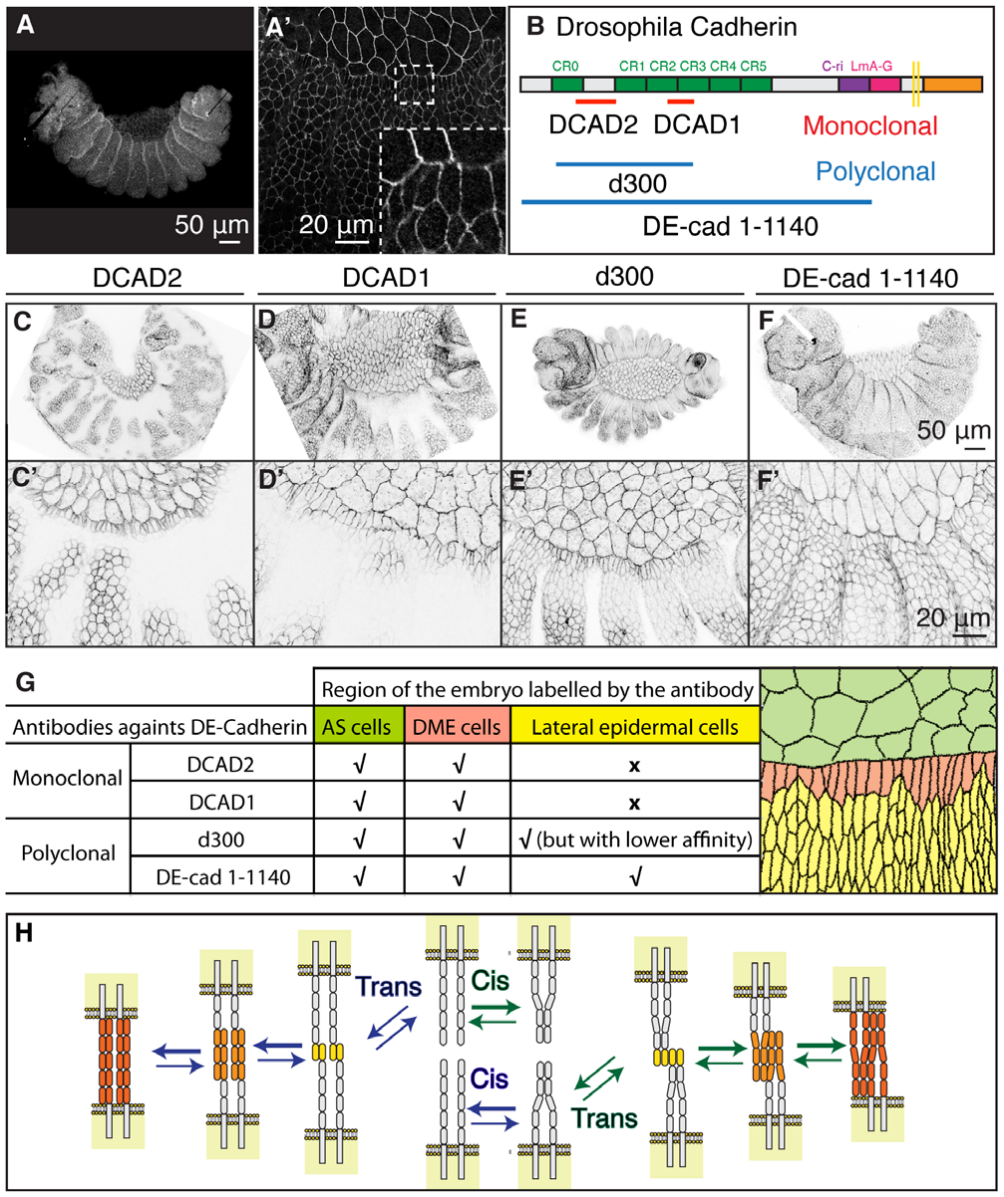
Antibodies against different epitopes of DE-Cadherin bind differently along the epidermis. (A,A′) Hand-devitellinized embryos fixed and then incubated with DCAD2 at 0′ chase. (B) Structure of *Drosophila* E-Cadherin depicting the epitopes recognized by the antibodies used in this study [41], [42], [43]. (C–F) Confocal z-projections of Ubi-DE-CadherinGFP embryos pulsed with DCAD2, DCAD1, d300 and DE-cad 1-1140 antibodies, fixed without chase. (G) Summary of anti-DE-Cadherin antibodies binding patterns in AS and epidermal cells. (H) Current models for interactions between Cadherins (C, adapted from Leckband and Prakasam, 2006).

In contrast with the standard immunostaining protocols, which result in a homogeneous binding of DCAD2 to the epidermis, we incubate the embryos with the antibodies before fixation, which results in a patterned DCAD2 labelling of the epidermis. In fact, fixation after hand-devitellinization but before antibody incubation also disrupts the pattern (Figure 3A,A′). Formaldehyde, which was used to fix the embryos, crosslinks proteins during the process of fixation, that can lead to artefacts such as chemical modification of proteins, which then can affect the interaction of the antibody with the antigen [32]. Therefore, the pattern observed with DCAD2 could be associated with a particular epitope or form of DE-Cadherin that is disrupted upon fixation.

To investigate whether there are differences in the extracellular domain of native DE-Cadherin along the epidermis we used the same assay with antibodies against different regions of the DE-Cadherin extracellular domain (Figure 3B). Another monoclonal antibody, DCAD1, bound to the AS, DME cells and more ventral epidermal cells (Figure 3C,C′). The DCAD1 antibodies also recognized patches of the lateral epidermis. The polyclonal antibody d300 bound to all the epidermal cells, but with different affinities across the epidermis (Figure 3E,E′); the lateral epidermis, which was not labelled with DCAD2, was weakly labelled by d300. On the other extreme, a polyclonal antibody generated against the entire extracellular domain of DE-Cadherin, DE-cad 1–1140, labelled all epidermal cells homogeneously, though with a lower affinity when compared with the other antibodies against DE-Cadherin (Figure 3F,F′).

These results indicate the existence of a spatial pattern of accessibility to the extracellular domain of DE-Cadherin in the epidermis at stages 13–15 (Figure 3G). These differences could result from different DE-Cadherin homophilic binding states, from different organization of DE-Cadherin at the cell surface or binding to other proteins at the cell surface.

We do not present direct data showing that these antibodies recognize different DE-Cadherin forms, nevertheless there are examples of antibodies that bind to different conformations of molecules involved in adhesion [33], [34], [35]. Furthermore, there is evidence for different configurations of Cadherin at the cell surface (Figure 3H) [1], [36], [37]. During the establishment of Cadherin mediated adhesion, dimerization of Cadherin molecules precedes *trans* interactions, but the lateral dimers can dissociate to form adhesive *trans*-homophilic bonds or remain dimerized in the *trans* interactions. Initially, N-terminal domain interactions are thought to have an important role in adhesion and binding selectivity in the *trans* interactions. However, these adhesive complexes can progress to associations involving further ectodomains that strengthens the adhesive bonds [36]. Since our results suggest that different regions of the extracellular domain of DE-Cadherin are more exposed in certain regions of the epidermis, we propose that the different antibodies recognize DE-Cadherins engaged in binding that involves a different number of extracellular domains. This also agrees with the existence of different pools of Cadherin with different adhesive properties as shown in the *Drosophila* epidermis [38].

### Genetic reduction of DE-Cadherin increases DCAD2 binding to epidermal cells

The observed surface pattern of DCAD2 binding correlates with different cell behaviours during DC [18]. To experimentally assess the functional role of the observed DCAD2 pattern along the epidermis, we performed the same assay in *shg^R64^* zygotic mutant embryos, which only have the maternal contribution of DE-Cadherin [18]. We reasoned that if the differences observed were linked to differential engagement in adhesion, altering the levels of Cadherin would alter this balance and therefore the pattern.

**Figure 4:**
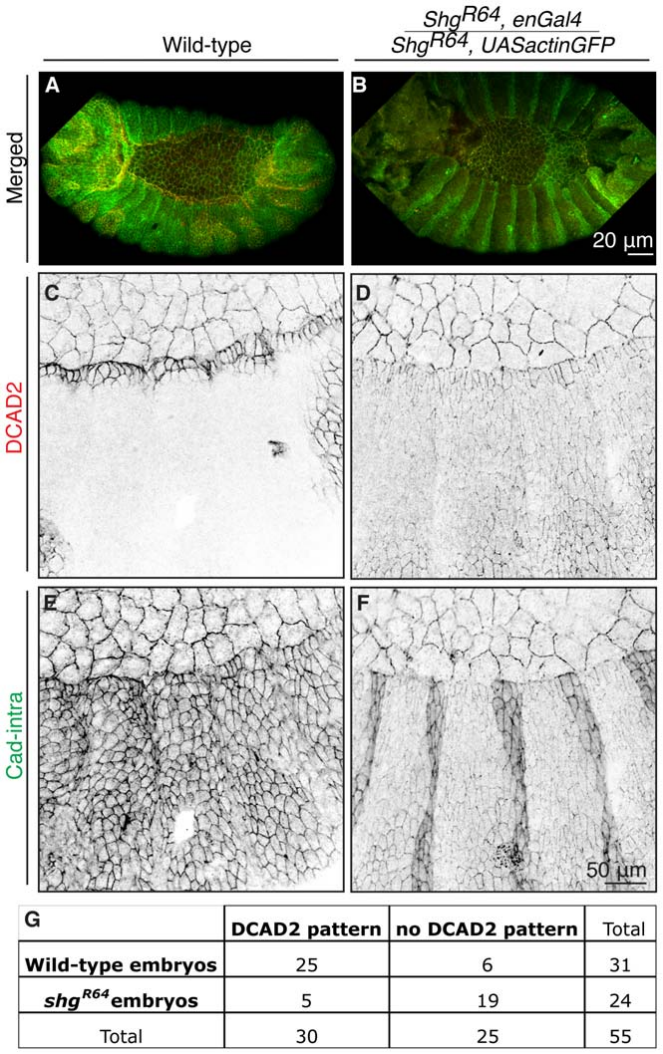
Genetic Reduction of DE-Cadherin increases DCAD2 binding to epidermal cells. Projection of confocal z-sections of wild-type (A, C, E) and *shg^R64^* homozygous mutant (B, D, F) embryos pulsed with DCAD2 at 0′ chase. *Shg^R64^* homozygous mutant embryos result from a cross between *shg^R64^,enGal4* and *shg^R64^,UASactinGFP*, therefore a stronger staining on the engrailed domain is detected in F. (G) Contingency table with the number of embryos exhibiting DCAD2 labelling pattern in wild-type embryos and *shg^R64^, UASactinGFP/shg^R64^, enGal4* embryos. The χ^2^-test revealed that the differences between wild-type and mutant embryos are extremely statistically significant (p<0.0001).

Cad-intra antibody (Figure 4 A,E) shows that the levels of Cadherin in wild-type are homogeneous along the epidermis but DE-Cadherin is detected differently on the extracellular domain by DCAD2 (Figure 4C). In the *shg^R64^* mutant, Cad-intra antibody clearly shows that the total levels of DE-Cadherin are lower than in wild-type (Figure 4B,F), which explains why DCAD2 antibody labelling is weak in the epidermis (Figure 4D). Interestingly, in *shg^R64^* mutant embryos DCAD2 antibody binds to DE-Cadherin in all epidermal cells (Figure 4D). We quantified the number of embryos exhibiting the DCAD2 labelling pattern in wild-type embryos and *shg^R64^, UASactinGFP*/*shg^R64^, enGal4* embryos, applied the χ^2^-test and found that the differences between wild-type and mutant embryos are statistically highly significant (p<0.0001; Figure 4G). In this attempt to manipulate the adhesive strength, lowering the amount of DE-Cadherin levels in the embryo could have led to epitope exposure and higher antibody accessibility, nevertheless when we tried an antibody against FasII, a component of the Septate Junctions that lies below the AJs, the binding was homogeneous (not shown), suggesting that the different binding of DCAD2 in wild-type or in the *shg^R64^* mutants is not result of different accessibility of the antibody to the protein epitope.

Our interpretation for the homogeneous binding of DCAD2 to all epidermal cells in the *shg^R64^* mutants is that in the mutant all DE-Cadherin is engaged in strong homophilic adhesion, to compensate for DE-Cadherin reduced levels and to avoid epidermal cells from falling apart. Accordingly with this consideration DCAD2-labelled-DE-Cadherin would be engaged in stronger adhesion. Although, there is no direct data that shows whether the differences in antibody binding reflect strong Cadherin-Cadherin interactions or weak Cadherin-Cadherin interactions, the results obtained in *shg^R64^* mutant suggest that DE-Cadherin exists in different forms that confer different adhesive strengths during DC in the *Drosophila* embryo.

**Figure 5:**
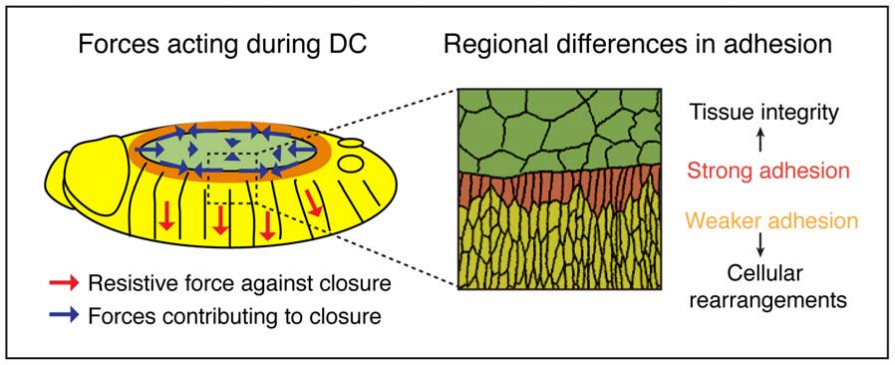
Forces driving DC. AS contraction, actin purse string and zippering contribute positively to closure, in contrast to the resistive force exerted by the epidermis . Model of regional differences in adhesion along the epidermis.

Downregulation of Cadherin mediated adhesion and changes of adhesive activity with no detectable changes in the levels of Cadherin were observed during *Xenopus laevis* development. Importantly, this change in Cadherin activity also altered the binding of antibodies to native Cadherin [39], [40]. Our results suggest that these adhesive Cadherin properties are conserved and provide direct evidence for the first time for a spatial cellular organization of Cadherin during a morphogenetic process. The pattern observed along the epidermis correlates with differential cell behaviour during DC. The DME cells are attached to the AS, remain tightly bound to each other and bear most of the mechanical stress of the process (Figure 5A). Therefore, they have strong staining, the stronger the closer to the LE. The lateral epidermal cells, that undergo continuous cell rearrangement and might be in a more fluid phase, show less staining than DME cells and the AS (Figure 5B). Moreover, the binding of DCAD2 to the AS is similar to the DME cells and cell intercalation has not been observed in the AS [17]. Finally, DE-Cadherin level reduction results in a more homogeneous binding and we suggest that in this situation the little DE-Cadherin available is engaged in stronger adhesion. This would implicate that in wild-type DE-Cadherin molecules in the lateral epidermis, that are not recognized by DCAD2, are engaged in weaker adhesion. Altogether, our results suggest that structural differences in the extracellular domain of Cadherin can mediate differential cell adhesion during development, allowing distinct cell behaviour required for morphogenesis.

## Supporting Information

Figure S1 **Validation of the live pulse-chase assay in embryos.** Pulse-chase assays with 0′ chase (first raw) and 30′ chase (second raw) were performed with antibodies against the extracellular domain of DE-Cadherin (A,B), the intracellular domain of Notch (A′,B′) and Scribble, an intracellular protein (A″,B″). Arrowheads show intracellular puncta positive for DCAD2 that result from endocytosis occurred during the 30′ of chase (B). The intracellular antibodies (against Notch-intra and Scribble) were not able to access the interior of the cell. In the third raw, embryos were fixed and permeabilized before incubation with the referred antibodies; under these conditions the antibodies against intracellular epitopes in Notch (C′) and Scribble (C″), can bind and reveal patterns of expression. Confocal sections from the z-stack projected in B (5 µm along the z-axis).
(TIF)

Figure S2 **Pulse-chase assay in ubi-DE-CadherinGFP embryos with DCAD2 and Notch-extra antibodies.** Pulse-chase assay of DE-Cadherin and Notch in ubi-DE-CadherinGFP embryos with DCAD2 and Notch-extra antibodies. After 30′ of chase at RT the DCAD2 pattern is still maintained (C, C′), but Notch levels continue to decrease in the cell membrane of the AS and epidermis (D, D′). The vesicles of Notch tend to be bigger and more basal (D′,D″). With 1 hour of chase at RT, DCAD2 is still present at the membrane of AS and DME cells and also in large cytoplasmic vesicles (G,G′,G″). Notch is cleared from the membrane and the number and size of vesicles is greatly reduced (H,H′,H″). (I) Quantitative comparison of DCAD2 and Notch labelling at the cell membrane of LE cells over time. A significant difference occurs in Notch between 0′ chase and 10′ chase (p<0.01, n_0′_ = 30 and n_10′_ = 30) and 0′ chase and 30′ chase (p<0.01, n_0′_ = 30 and n_30′_ = 20) but not in DCAD2 (error bars show the SD).
(TIF)

Figure S3 **DCAD2 pattern is also observed in wild-type** ***Drosophila*** **embryos.** Using the standard staining protocol for *Drosophila* embryos, in which fixation and permeabilization precedes antibody incubation, DCAD2 binds homogeneously to the epidermis and AS, regardless of the DC stage (A–D′). The pattern of DCAD2 observed in ubi-DE-CadherinGFP expressing embryos is also observed in wild-type embryos at different time points of the pulse-chase (E′, F′,G′).
(TIF)

Movie S1 **Time-lapse of a hand-devitellinized Ubi-DE-CadherinGFP embryo.**
(AVI)
