# Supplementary figures and images for: Patterned Cell Adhesion Associated with Tissue Deformations during Dorsal Closure in Drosophila
Source: PLoS One. 2011 Nov 4;6(11):e27159. doi: 10.1371/journal.pone.0027159 (PMC3208594; doi:10.1371/journal.pone.0027159)

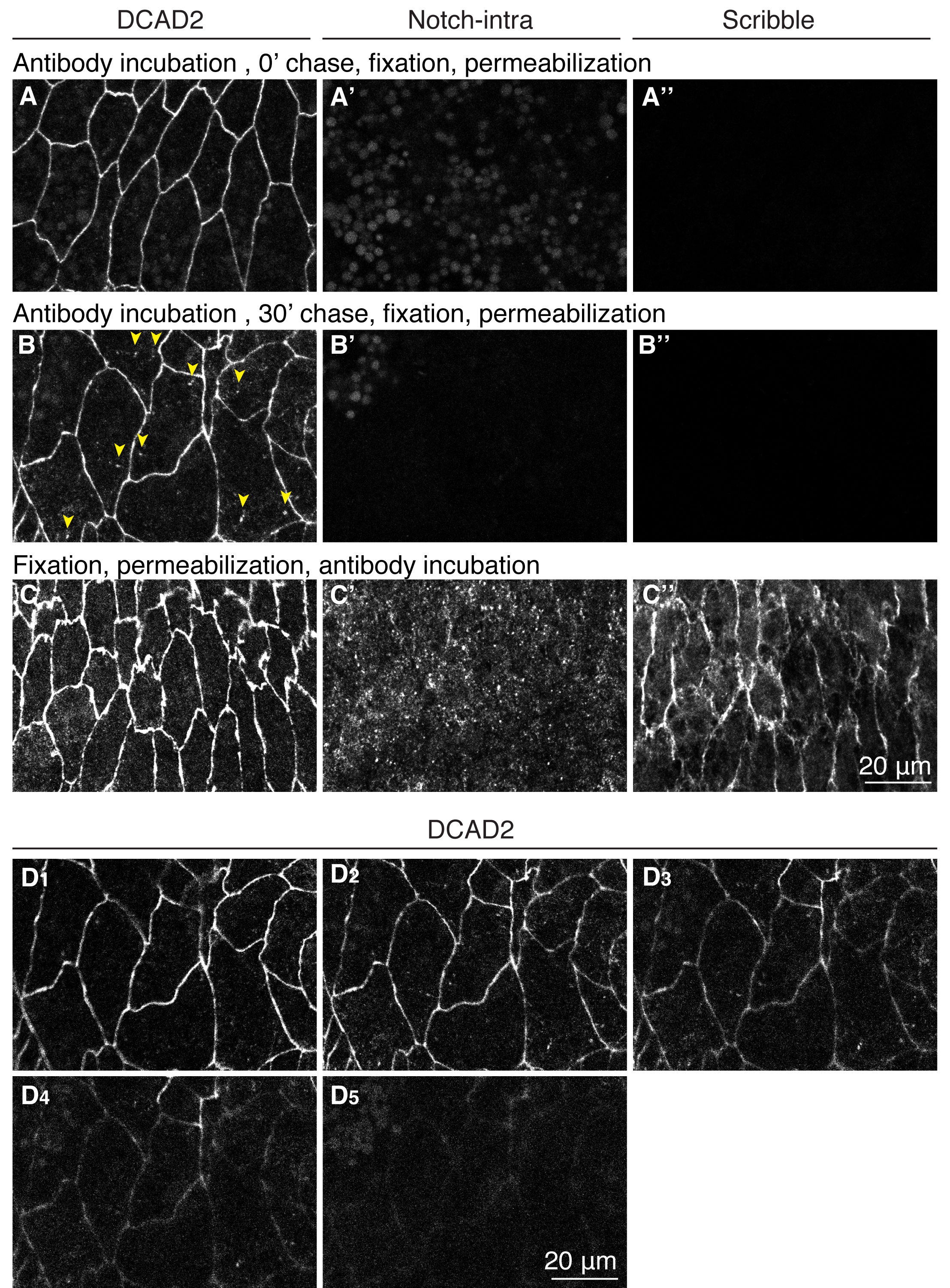

Supplement: Figure S1 — Validation of the live pulse-chase assay in embryos. Pulse-chase assays with 0′ chase (first raw) and 30′ chase (second raw) were performed with antibodies against the extracellular domain of DE-Cadherin (A,B), the intracellular domain of Notch (A′,B′) and Scribble, an intracellular protein (A″,B″). Arrowheads show intracellular puncta positive for DCAD2 that result from endocytosis occurred during the 30′ of chase (B). The intracellular antibodies (against Notch-intra and Scribble) were not able to access the interior of the cell. In the third raw, embryos were fixed and permeabilized before incubation with the referred antibodies; under these conditions the antibodies against intracellular epitopes in Notch (C′) and Scribble (C″), can bind and reveal patterns of expression. Confocal sections from the z-stack projected in B (5 µm along the z-axis). (TIF) [file pone.0027159.s001.tif]

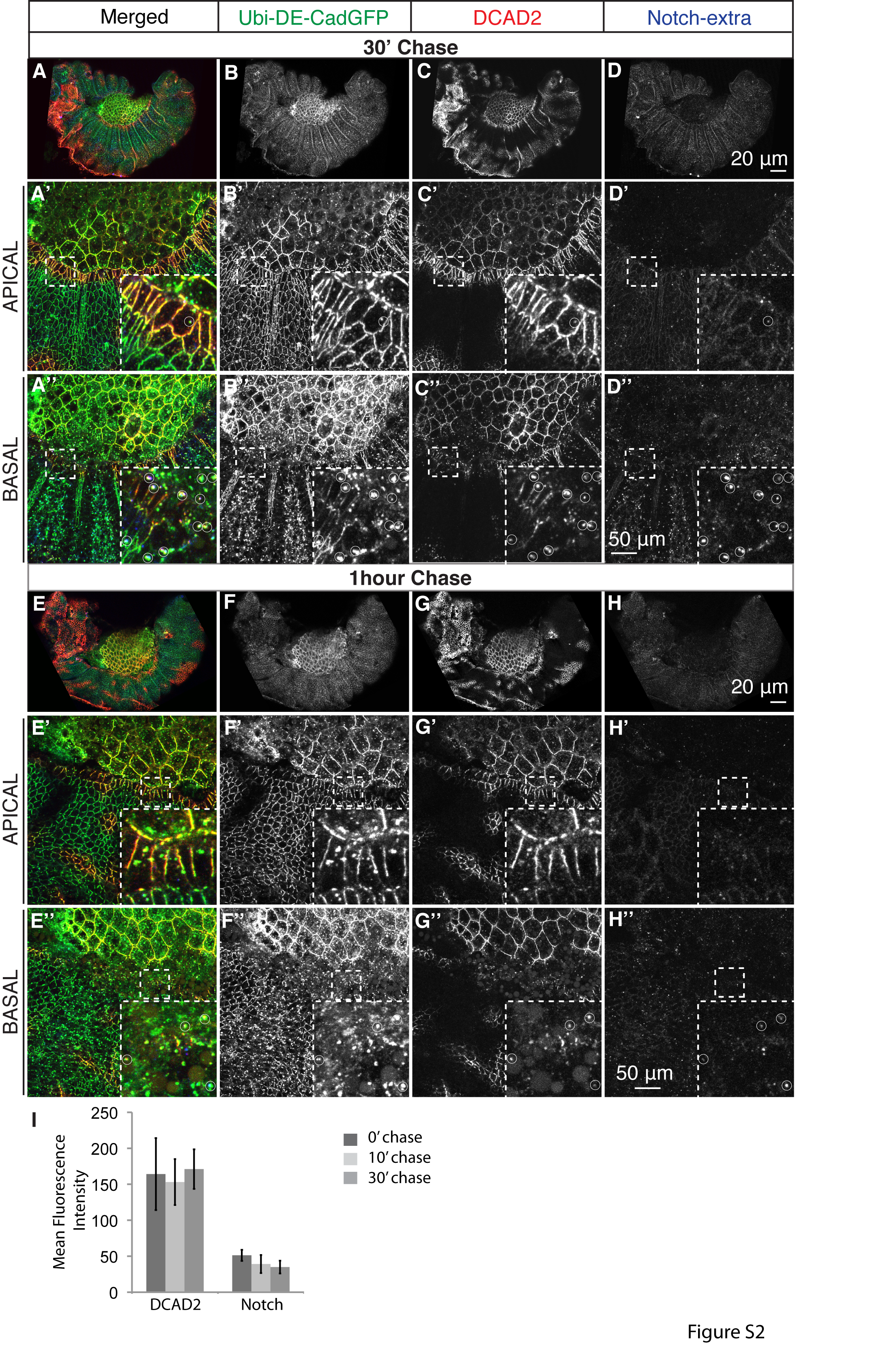

Supplement: Figure S2 — Pulse-chase assay in ubi-DE-CadherinGFP embryos with DCAD2 and Notch-extra antibodies. Pulse-chase assay of DE-Cadherin and Notch in ubi-DE-CadherinGFP embryos with DCAD2 and Notch-extra antibodies. After 30′ of chase at RT the DCAD2 pattern is still maintained (C, C′), but Notch levels continue to decrease in the cell membrane of the AS and epidermis (D, D′). The vesicles of Notch tend to be bigger and more basal (D′,D″). With 1 hour of chase at RT, DCAD2 is still present at the membrane of AS and DME cells and also in large cytoplasmic vesicles (G,G′,G″). Notch is cleared from the membrane and the number and size of vesicles is greatly reduced (H,H′,H″). (I) Quantitative comparison of DCAD2 and Notch labelling at the cell membrane of LE cells over time. A significant difference occurs in Notch between 0′ chase and 10′ chase (p<0.01, n0′ = 30 and n10′ = 30) and 0′ chase and 30′ chase (p<0.01, n0′ = 30 and n30′ = 20) but not in DCAD2 (error bars show the SD). (TIF) [file pone.0027159.s002.tif]

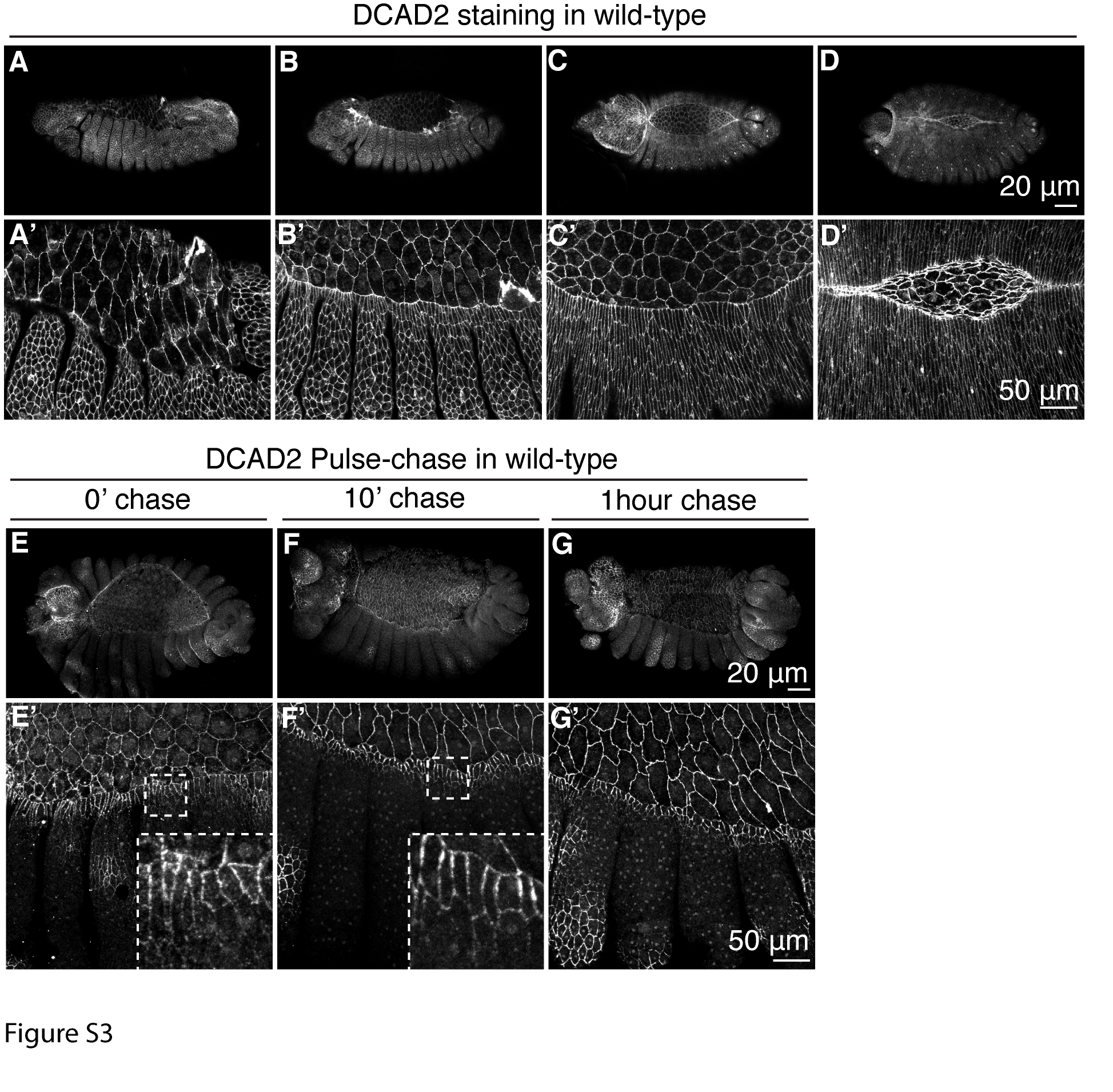

Supplement: Figure S3 — DCAD2 pattern is also observed in wild-type Drosophila embryos. Using the standard staining protocol for Drosophila embryos, in which fixation and permeabilization precedes antibody incubation, DCAD2 binds homogeneously to the epidermis and AS, regardless of the DC stage (A–D′). The pattern of DCAD2 observed in ubi-DE-CadherinGFP expressing embryos is also observed in wild-type embryos at different time points of the pulse-chase (E′, F′,G′). (TIF) [file pone.0027159.s003.tif]
